# An Atypical Cardiac Manifestation of Fabry Disease from a Novel Pathological Variant on the GLA Gene

**DOI:** 10.7759/cureus.7262

**Published:** 2020-03-13

**Authors:** Luay Sarsam, Amy Arouni, Toufik Mahfood Haddad, Cherry O Onaiwu, Christopher Erickson

**Affiliations:** 1 Cardiovascular Disease, Arnot Ogden Medical Center, Elmira, USA; 2 Cardiology, Veterans Affairs Nebraska-Western Iowa Health Care System, Omaha, USA; 3 Cardiology, Creighton University School of Medicine, Omaha, USA; 4 Internal Medicine, Houston Methodist Hospital, Houston, USA; 5 Pediatrics and Internal Medicine, University of Nebraska College of Medicine, Omaha, USA

**Keywords:** cardiac phenotype, left ventricular hypertrophy, fabry disease

## Abstract

Fabry disease (FD) is one of the most common lysosomal storage disorders and is caused by an X-linked progressive inborn error of metabolism in the alpha-galactosidase A (α-Gal A) gene. This leads to intracellular accumulation of glycosphingolipids, mainly globotriaosylceramide (Gb3), throughout the body. The impact of this accumulation is seen across multiple cell lines and therefore can cause multisystem organ dysfunction. The phenotype of FD results from variants on the GLA gene which codes for α-Gal A production, and variants on this gene have been shown to be strongly related to unexplained or idiopathic cardiovascular disorders. This report describes a 36-year-old Caucasian male found to have left ventricular hypertrophy (LVH) followed by genetic testing because of his family history of sudden cardiac death which revealed a variant of unknown significance for the GLA gene. Further measurement of α-Gal A leukocyte activity showed low levels, which was diagnostic for FD. The index patient had an unusual non-classic phenotype in that his sole presenting symptom was asymptomatic LVH, he presented early, and had low α-Gal A leukocyte activity. Early detection and prompt treatment with enzyme replacement therapy can improve outcomes and decrease mortality. In the absence of known risk factors, non-classical FD should be strongly considered in patients with unexplained LVH and a family history of sudden cardiac death at a young age.

## Introduction

Fabry disease (FD) is one of the most common lysosomal storage disorders and is most prevalent in Caucasian males [1]. It is the result of an X-linked progressive inborn error of metabolism in the alpha-galactosidase A (α-Gal A) gene which leads to intracellular accumulation of glycosphingolipids, mainly globotriaosylceramide (Gb3), throughout the body [2].The impact is widespread and affects multiple cell lines including the kidneys, the nervous system, and the heart, and therefore can cause irreversible tissue ischemia and fibrosis which may lead to multisystem organ dysfunction [3]. The phenotype of FD results from variants on the GLA gene which codes for α-Gal A production. Variants on this gene have been shown to be strongly related to unexplained or idiopathic cardiovascular disorders [2].

## Case presentation

The index patient is a 36-year-old Caucasian male who underwent a screening transthoracic echocardiogram (TTE) at age 31 years after his older brother had a sudden cardiac death. The patient has a past medical history of mild hypertension successfully treated with clonidine, nicotine dependence, and post-traumatic stress disorder. Social history is significant for a 27-pack-year smoking history and rare alcohol use. Family history is significant for an older brother with Wolff-Parkinson-White (WPW) syndrome and sudden cardiac death at age 33 years. Autopsy showed evidence of hypertrophic cardiomyopathy (HCM) of unknown etiology. Family history is also significant for multiple maternal relatives with premature coronary artery disease and myocardial infarction. 

The index patient’s initial TTE at age 31 years showed preserved left ventricular ejection fraction (LVEF), normal diastolic function, no valvulopathy, and no left ventricular hypertrophy (LVH) with a septum measurement of 8.1 mm and a posterior wall measurement 10.3 mm in diastole. The patient was referred to cardiology outpatient clinic for further evaluation due to increased risk for cardiac pathology because of his family history. On presentation, he did not exhibit any signs or symptoms of active cardiac disease. He denied chest pain, palpitations, dyspnea, orthopnea, lightheadedness, dizziness, and syncopal or presyncopal episodes.

Vital signs showed temperature 99.2°F, pulse 70 beats per minute (bpm), respiratory rate 18 breaths per minute, and blood pressure 106/70 mmHg. On physical examination, he had a normal heart rate and regular rhythm, normal heart sounds, no murmurs, no evidence of jugular venous distention, and no lower extremity edema. There were no signs of neurological or dermatological disease. A complete metabolic panel and complete blood count were within normal limits. Observation and routine surveillance was recommended.

**Figure 1:**
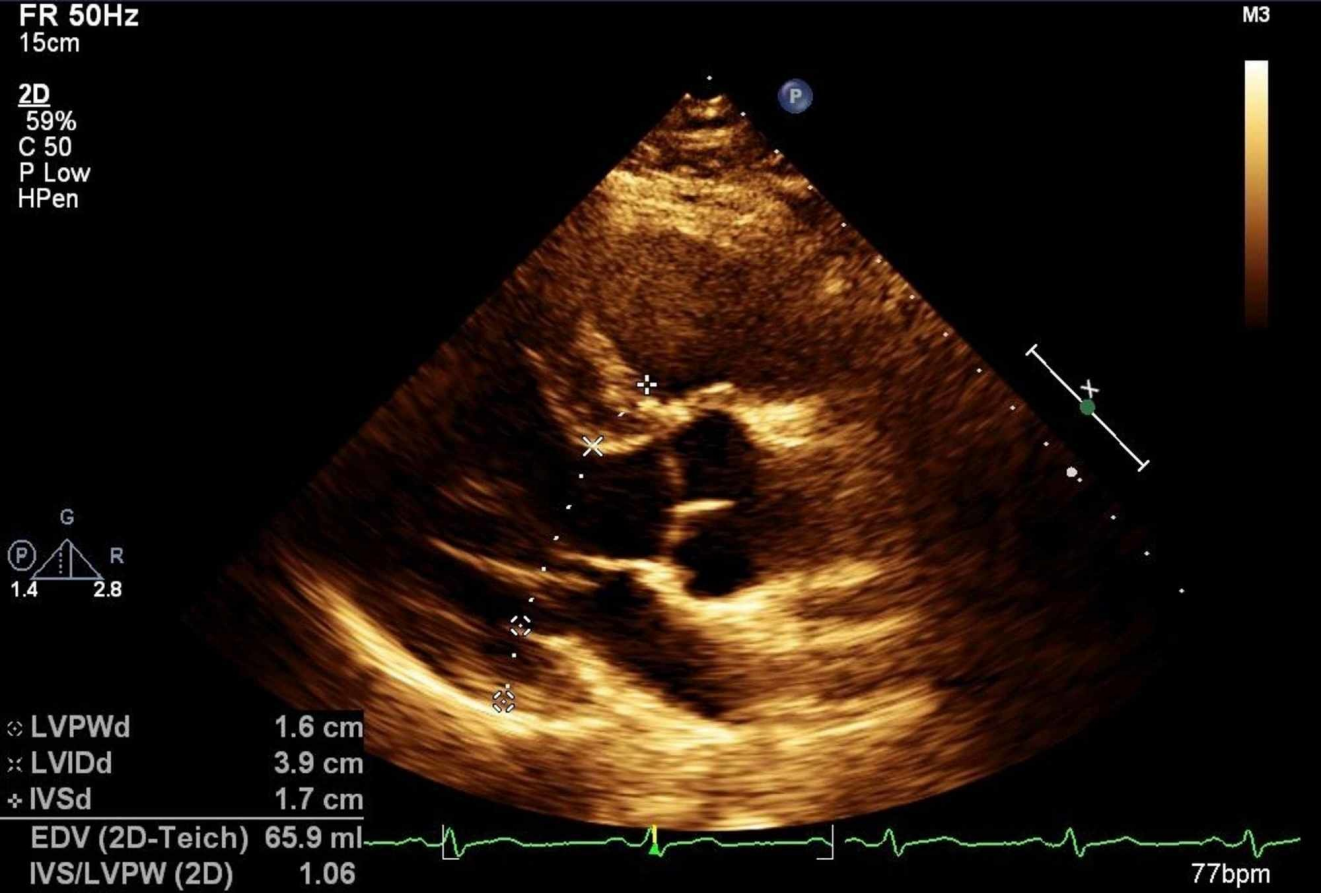
TTE demonstrating moderate LVH with septal wall thickness of 18 mm and posterior wall thickness 17 mm. Chordal SAM is observed. TTE, transthoracic echocardiogram; LVH, left ventricular hypertrophy; SAM, systolic anterior motion.

The patient had another TTE at age 33 years which showed mild LVH as his interventricular septum measured 12 mm and the posterior wall measured 13 mm in diastole. There was a normal resting left ventricular outflow tract (LVOT) gradient, but there was chordal systolic anterior motion (SAM). End diastolic dysfunction was not present at that time. More recently, at age 36 years during routine cardiology follow-up, the patient’s review of systems was now positive for rare episodes of orthostatic dizziness and progressive dyspnea with moderate exertion. The patient had a repeat TTE at that time which showed evidence of progressive, now moderate LVH. In diastole, the septum measured 17 mm which was increased from 12 mm on previous imaging and the posterior wall measured 16 mm which was increased from 13 mm on previous imaging (Figure 1). There was chordal SAM. Grade II diastolic dysfunction was present with preserved LVEF of 65%.

Further diagnostic testing included an electrocardiogram (EKG) which showed normal sinus rhythm but no unusual voltage for LVH. Echocardiogram exercise stress test did not show evidence of ischemia or malignant arrhythmias with maximal stress. LVOT gradient of 33 mmHg was noted in the recovery phase with a heart rate of 106 bpm. No symptoms of chest pain or angina were noted. Duke protocol risk stratification was low. Cardiac monitoring for two weeks showed a predominant underlying sinus rhythm. No significant non-sustained ventricular tachycardia (VT) or sustained VT was noted.

**Figure 2:**
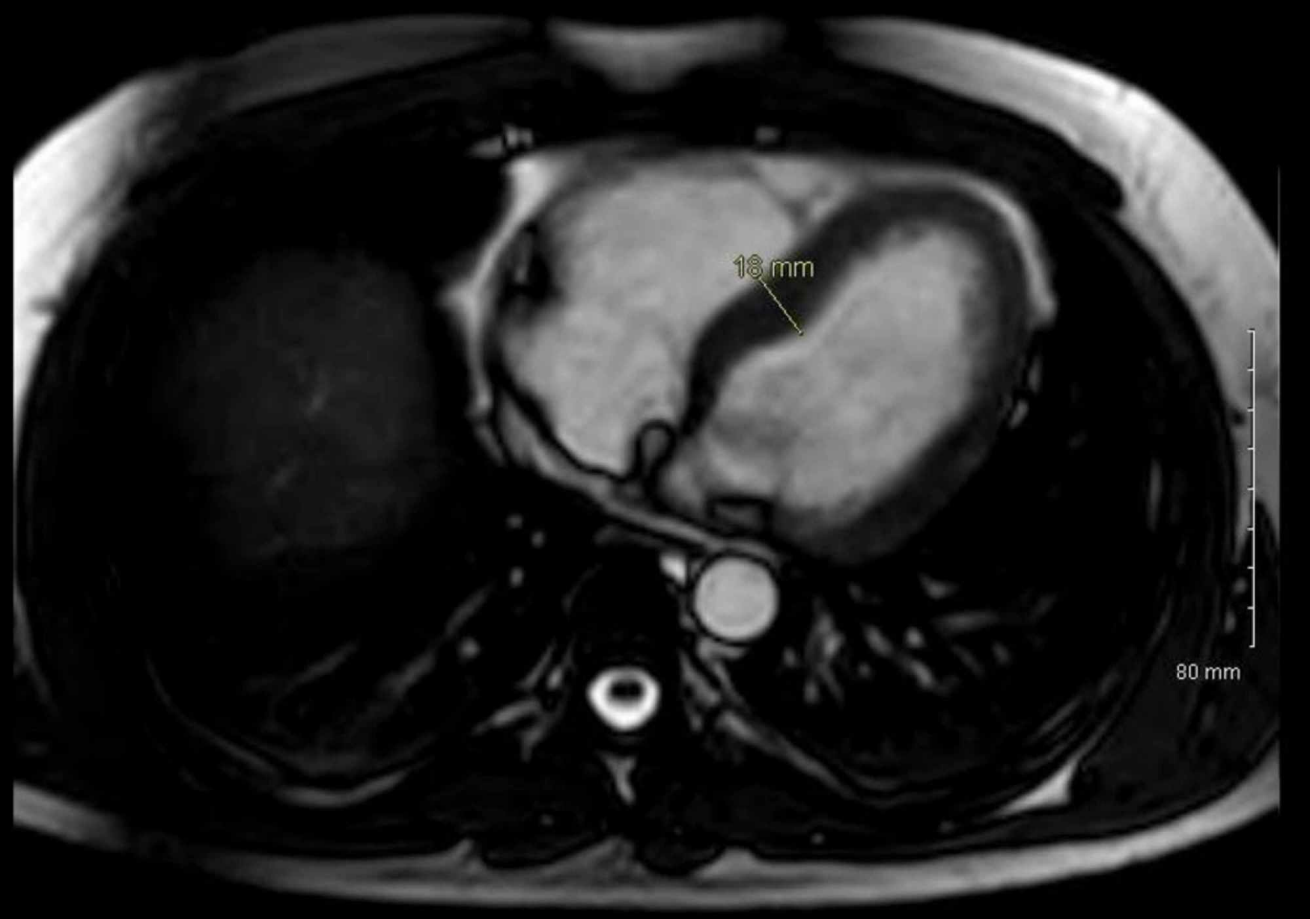
Cardiac MRI demonstrating LVH with septal thickness of 18 mm. LVH, left ventricular hypertrophy.

With the diagnosis of HCM along with the history of sudden cardiac death of his first-degree relative, he was referred for genetic counseling at the Genetic-Arrhythmia Clinic for further evaluation. Cardiac MRI showed HCM with a maximum interventricular septal dimension of 18 mm (Figure 2) and a posterior wall dimension of 17 mm. There was no evidence of LVOT obstruction. There was a hyperdynamic left ventricular (LV) function with an ejection fraction of 64% and a normal right ventricle size, wall thickness, and systolic function. His genetic testing demonstrated a variant of unknown significance (VUS) for the GLA gene (c.574 A>T). Further measurement of α-Gal A leukocyte activity showed low levels of 1.5 nmol/h/mg (lab reference range ≥ 23.1 nmol/h/mg), which was diagnostic for FD. The patient was subsequently initiated on beta-blocker therapy with plan for serial cardiac MRI to monitor for progression of LVH. After genetic counseling and discussion regarding the initiation of enzyme replacement therapy (ERT), the patient opted to be monitored for further progression and/or symptoms prior to initiation due to potential adverse events of initiating therapy. 

## Discussion

Although seen in multiple ethnic groups, FD mainly affects Caucasian males like the index patient with studies showing a frequency as high as one in 1,500 in some countries [1,4]. The incidence reportedly ranges up to one in 117,000, which may be a gross underestimate as studies have shown a higher than expected prevalence in multiple populations through newborn screening for deficiencies in α-Gal A activity [1,4,5]. The discrepancy is thought to be a result of different phenotypes as in the index patient whose genetic testing revealed a VUS on GLA resulting in decreased leukocyte α-Gal A activity causing this phenotype of FD [2].

Clinical manifestations of classic FD can present in early childhood and affect multiple organ systems. Patients can present with a multitude of vague symptoms making diagnosis difficult, including pain, gastrointestinal upset such as diarrhea and abdominal pain, angiokeratoma, hypohidrosis, visual disturbances due to corneal deposits, fatigue, and more commonly neurological, renal, and cardiac abnormalities [5]. Typical cardiac findings include LVH, ascending aorta dilatation, valvular structural changes, EKG abnormalities, and arrhythmias. Female heterozygotes can also be impacted with a wide range of clinical signs and symptoms which can be mild to severe in nature. This is thought to be a result of skewed X-chromosome inactivation [5].

Studies show that the severe and early onset systemic manifestations of classical FD are a result of absent or low levels of α-Gal A activity [2]. In such patients cardiac involvement usually becomes obvious between 20 and 40 years of age; however, in patients such as the index patient with a non-classic phenotype of FD, the major manifestations of classic FD may be absent, and clinical complications such as kidney failure, cerebrovascular accident, and cardiomyopathy usually present in the fourth to sixth decade and may be confined to a single organ [2,5-7]. A recent retrospective case series analyzing multiple unrelated families with a GLA VUS similar to that in the index patient demonstrated that patients with classic FD had no or negligible levels of α-Gal A activity, while non-classic FD patients had significant residual levels of activity [7].

Studies suggest that variations of FD can be strongly related to unexplained or idiopathic cardiovascular disorders, and patients who present with cardiac manifestations of FD without overt systemic involvement are known to have a genetic variant that may be specific for cardiac pathology [2,8]. Three studies at different centers conducted on 2,084 men with unexplained LVH found that 0.9%-3.92% of those men had FD diagnosed either by biopsy or measurement of α-Gal A activity [8-10]. Also, a European multicenter cross-sectional study by Elliott et al. found that 0.5% of its 1,386 patients with unexplained LVH had GLA mutations associated with FD [11].

The accumulation of Gb3 in the myocardium can cause hypertrophy that is difficult to distinguish from typical HCM on imaging. The criterion for the diagnosis of LVH is as follows: echocardiogram findings of LV mass > 115 g/m^2^ or a ventricular-septum and/or posterior-wall thickness in males of at least 13 mm [12,13]. The index patient presented with LVH with an interventricular septal dimension of 18 mm (Figure 2), a posterior wall dimension of 17 mm, and a LV mass of 262 g. He has a history of mild hypertension, which is one of the most common causes for cardiac hypertrophy; however, his hypertensive heart disease was treated, well controlled, short in duration (<10 years), and did not show evidence of end organ damage. Also, LV wall thickness from hypertension alone does not usually exceed 16 mm and is not associated with a LVOT gradient or SAM of the mitral valve leaflet [14]. Highly trained athletes according to some studies can have LV wall thickness up to 16 mm due to athlete’s heart. In these cases, a distinction between physiological heart and pathological heart can be made through echocardiogram or cardiac MRI [12,13]. Some females can be heterozygous for the Fabry mutation, and due to skewed X-chromosome inactivation they may vary greatly in signs and symptoms of FD [5].

FD can be diagnosed biochemically through deficient activity of α-Gal A in plasma or leukocytes on enzymatic assay in males, via a less reliable measurement of plasma and/or urine Gb3 levels, or definitively through detection of a GLA mutation on genetic analysis [5]. The GLA variant in this patient along with the profound decreased α-Gal A activity is evidence that this variant is pathogenic and should no longer be classified as a VUS.

Management of FD consists of supportive care including pain management, anti-inflammatory medication, and management of systemic sequelae, including dialysis, antihypertensive therapy, and anticonvulsants. Treatment includes enzyme replacement therapy with recombinant α-Gal A. There are currently two drugs on the market, agalsidase alfa and agalsidase beta (both are recombinant human α-Gal A), the latter of which is approved by the United States Food and Drug Administration. These enzymes work to clear microvascular endothelial deposits of Gb3 [15]. Randomized controlled and open-label studies have shown that initiation of ERT in patients with cardiac manifestations of FD resulted in decreased LV mass, decreased LV posterior wall and septal wall thickness, improved cardiac conduction, reduced heart rate, reduced LV end diastolic volume, and improved LV function [15,16]. Initiation of ERT prior to evidence of myocardial fibrosis produced significant reductions in LVH [16].

Our patient is unusual in that he has a non-classic phenotype of FD, despite an extremely low α-Gal A activity level (1.5 nmol/h/mg), which has not been described previously in the literature. Further, patients with FD usually present with multiple clinical manifestations of FD, but this patient’s sole presenting symptom was LVH (phenocopy of HCM). He had no other manifestations of FD; the diagnosis was suspected only based on his progressive LVH. To our knowledge, there are no other reported cases of FD where the sole clinical manifestation is LVH. This patient presented early in his course (in his third decade) when patients with non-classic FD usually present in their fourth to sixth decade. Finally, the index patient’s clinical course is unusual in that genetic evidence of a mutation in the α-Gal A was discovered prior to assessment of α-Gal A levels.

It is likely that his older brother who suffered an unexplained sudden cardiac death in his third decade may have also suffered from FD as post-mortem analysis did show evidence of LVH. Studies show an association between cardiac arrhythmias in FD and sudden cardiac death [17]. The mechanism is not completely understood; however, evidence suggests a possible relation to the accumulation of glycosphingolipids, myocyte hypertrophy, and interstitial hypertrophy in the left ventricle [17]. It is noteworthy that, while alive, his brother was diagnosed with WPW syndrome and underwent cardiac ablation. There are a few case reports that show a rare association between FD and WPW, which is thought to result from glycolipid deposition in the conducting system around the atrioventricular node [18]. The presence of WPW cannot be ruled out as a potential cause of death particularly in light of the brother’s history of an ablation.

## Conclusions

The patient that is the subject of this report has a novel GLA variant, previously described as a VUS. There is now reasonable evidence that this variant should be classified as disease-causing or pathogenic. In addition, the phenotype of exclusive LVH exhibited by this patient is unique and previously not reported. Hence, isolated LVH in a young patient deserves a detailed evaluation and possible referral to a center specializing in HCM. In the absence of known risk factors, non-classic FD should be strongly considered in patients with unexplained LVH, a family history of sudden cardiac death at a young age, and a family pedigree suggesting X-linked inheritance. Extensive work-up and confirmation of diagnosis through genetic testing and/or biochemical analysis are critical as prompt initiation of ERT before the development of fibrosis can improve cardiac function, prevent deterioration in functional capacity, and decrease mortality.
